# Paracetamol in therapeutic dosages and acute liver injury: causality assessment in a prospective case series

**DOI:** 10.1186/1471-230X-11-80

**Published:** 2011-07-15

**Authors:** Mònica Sabaté, Luisa Ibáñez, Eulàlia Pérez, Xavier Vidal, Maria Buti, Xavier Xiol, Antoni Mas, Carlos Guarner, Montserrat Forné, Ricard Solà, José Castellote, Joaquim Rigau, Joan-Ramon Laporte

**Affiliations:** 1 Fundació Institut Català de Farmacologia, Universitat Autònoma de Barcelona, Hospital Universitari Vall d'Hebron, Barcelona, Spain; 2 Liver Unit, Hospital Universitari Vall d'Hebron, Barcelona, Spain; 3 Gastroenterology Service, Hospital Universitari Prínceps d'Espanya de Bellvitge, Hospitalet de Llobregat, Spain; 4 Liver Unit, Hospital Clínic, IDIBAPS, Barcelona, Spain; 5 Gastroenterology Service, Hospital de la Santa Creu i Sant Pau, Barcelona, Spain; 6 Gastroenterology Service, Hospital de Mútua, Terrassa, Spain; 7 Liver Section, Hospital Universitari del Mar, Universitat Autònoma de Barcelona, Spain; 8 Gastroenterology Service, Hospital General, Granollers, Spain

## Abstract

**Background:**

Acute liver injury (ALI) induced by paracetamol overdose is a well known cause of emergency hospital admission and death. However, there is debate regarding the risk of ALI after therapeutic dosages of the drug.

The aim is to describe the characteristics of patients admitted to hospital with jaundice who had previous exposure to therapeutic doses of paracetamol. An assessment of the causality role of paracetamol was performed in each case.

**Methods:**

Based on the evaluation of prospectively gathered cases of ALI with detailed clinical information, thirty-two cases of ALI in non-alcoholic patients exposed to therapeutic doses of paracetamol were identified. Two authors assessed all drug exposures by using the CIOMS/RUCAM scale. Each case was classified into one of five categories based on the causality score for paracetamol.

**Results:**

In four cases the role of paracetamol was judged to be unrelated, in two unlikely, and these were excluded from evaluation. In seven of the remaining 26 cases, the RUCAM score associated with paracetamol was higher than that associated with other concomitant medications. The estimated incidence of ALI related to the use of paracetamol in therapeutic dosages was 0.4 per million inhabitants older than 15 years of age and per year (99%CI, 0.2-0.8) and of 10 per million paracetamol users-year (95% CI 4.3-19.4).

**Conclusions:**

Our results indicate that paracetamol in therapeutic dosages may be considered in the causality assessment in non-alcoholic patients with liver injury, even if the estimated incidence of ALI related to paracetamol appears to be low.

## Background

Paracetamol is the most important pharmacological cause of acute liver failure (ALF) [1-5] and is the primary cause of overdose in the UK, USA and Scandinavian countries. It is thought to be safe in recommended doses, up to 4 g per day in adults. The public health impact of a paracetamol overdose has been evaluated in several epidemiological studies [1-3,5,6]. In a multicentric survey of ALF including hospitals attending approximately half the Spanish population, paracetamol was considered to be the cause of ALF in only 2.6% of 267 cases identified between 1992 and 2000, and the estimated incidence of ALF during the study period was of 1.4 cases per million inhabitants per year [7]. In contrast, liver injury after therapeutic dosages appears to be highly unusual and the mechanism accepted for the overdose liver injury has in fact been under discussion [8-10].

In a clinical trial in healthy volunteers who received 4 g of paracetamol daily, significant increases in alanine aminotransferase (ALT) were recorded [11]. The clinical significance of these results is unknown. In an epidemiological study a relative risk (RR) of acute liver injury (ALI) associated with paracetamol in therapeutic dosages, of 7.0 (99% CI 3.3-13.9) with an incidence rate of 2.4 cases per 100,000 person-years of exposure, was estimated [12].

The aim is to describe the characteristics of patients admitted to hospital with jaundice who had previous exposure to therapeutic dosages of paracetamol. An assessment of the causality role of paracetamol was performed in each case.

## Methods

### Case selection

Between January 1993 and December 1999, 32 cases of ALI in patients exposed to therapeutic dosages of paracetamol (4 g per day or less) were assembled as part of a multicentric prospective epidemiological study, with a systematic ascertainment of the incident cases of ALI, irrespective of the suspected cause [12].

A collaborating network including 12 hospitals covering a population of 2.7 × 10^6 ^inhabitants of 15 years of age or older was set up. To avoid any selection bias the patients were selected on the basis of their condition, rather than on the basis of suspected exposures. Cases of ALI were defined as jaundice with a total bilirubin level of ≥ 3 mg/dl and an acute increase in ALT of at least five times the upper limit of the normal range and/or an increase in alkaline phosphatase (AP) of at least twice the upper limit of the normal range. Patients with alcohol consumption greater than 50 g per day and/or with other possible causes of liver injury were excluded (the principal causes were serologically proven viral hepatitis, alcoholic liver disease, processes causing obstruction of bile flow and drug overdoses). The pattern of ALI was classified as hepatocellular, cholestatic or mixed, according to the International Consensus Meeting designations of ALI [13].

The patients were interviewed, after informed consent was given, using a structured questionnaire covering their past medical history, clinical course of the present condition, and detailed information on the use of medicines and exposure to environmental toxins in the previous three months. Clinical and laboratory data were also recorded.

As described, this was a population-based study, and thus allowed us to estimate the incidence of ALI related to paracetamol based on the cases defined as probably or possibly related to paracetamol exposure. The incidence on paracetamol users was estimated on the basis of drug consumption data. Confidence limits at 95% were used to obtain an approach to the precision of the estimated incidence.

The methods have been described in detail elsewhere [14].

### Causality assessment

Two authors assessed all the drugs taken by each patient in the three-month period before laboratory diagnosis in each case using the CIOMS/RUCAM scale [15]. At present the RUCAM algorithm is widely used for causality assessment of hepatotoxicity [16-19].

The method provides a score for each individual drug taken by a patient with ALI. The score allows the classification of each drug into five categories: "highly probable" (score > 8), "probable" (6 - 8), "possible" (3 - 5), "unlikely" (1 - 2) and "excluded" (< or = 0). When the time of onset is clinically incompatible, the drug is classified as "unrelated".

Each case was classified into one of five categories based on the score related to the causal role of paracetamol.

Details on the application of the seven items of the RUCAM scale were agreed on before the evaluation. Non-pharmacological possible causes of ALI were not present, because of stringent selection criteria (see Methods above). There were no cases reexposed to paracetamol after liver damage occurred. The cases presented in the results are those that paracetamol had a higher RUCAM score than the concomitant drugs.

In the 90's it was not compulsory (as it is nowadays) to have the approval of an appropriate ethics committee for observational studies in Spain. However, the study was approved by: CIRIT (Comisionat per a Universitats i Recerca, Generalitat de Catalunya) Accions Mobilitzadores (1995XT 00030) (Committee for Universities and Research, Government of Catalonia) Mobilization Resources, 1995XT 00030).

## Results

Up to December 31 1999, total follow up was 17,616,592 person-years and 126 patients fulfilling the inclusion criteria of ALI were identified. Drug exposures among these patients with ALI and comparative risks associated with various commonly used drugs have been reported on [12,14].

The RUCAM scale was applied to 32 cases exposed to paracetamol. In four cases paracetamol exposure started on the day the first symptoms of ALI (fever, abdominal pain, marked weakness, skin rash and/or pruritus, jaundice or choluria) appeared, and they were therefore classified as unrelated to paracetamol. In two cases the RUCAM score for paracetamol was < or = 2 and they were classified as unlikely. Of the remaining 26 cases, the score for paracetamol was higher than that for other concomitant drugs (7 cases). In 19 additional cases that took drugs that are known to be hepatotoxic agents, these drugs scored equal or higher than paracetamol. The different scores between the cases classified as "probable" and those classified as "possible" were mainly due to the differences in three items: time to onset, the course of the reaction after cessation of the drug, and previous information on the association between paracetamol exposure and liver injury, which in one case with a mixed pattern scored one point less.

Several features for the 7 cases (4 probable and 3 possible) with a paracetamol score higher than that of concomitant drugs are provided in table 1

**Table 1:** Patients classified as a probable or possible based on paracetamol score (RUCAM algorithm).

								Use of paracetamol					
											
Case	Sex/Age	Alcohol use (g/d)	ALT/AP	Bili	Pattern of ALI	Hypersensitivity	Outcome	Indication	Daily dose (mg)	Pattern of use	RUCAM Score	Concomitant drugs	RUCAM Score
**Probable (6 to 8)**													

1	F/58	43	15.85/1.74	1.36	HC	No	Tx	Osteoarthritis	650	Once a week for years till day before ALI.	8		
												Dexchlorpheniramine	7

2	F/82	0	15.32/1.95	10.41	HC	Thrombocytopenia	Recovered	Cold	650	Daily for 10 days before ALI.	7		
												Metamizol	6
												Dextromethorphan	6
												Chlorphenamine	6
												Indapamide	6
												Indomethacin	6
												Tocopherol	5

3	F/85	0.5	22.21/1.27	12.32	HC	Arthralgia, thrombocytopenia	Not recovered	Osteoarthritis	1950	Daily for 22 days before ALI.	7		
												Corticosteroids	6

4	F/25	24	383.33/2.35	3.64	HC	Eosinophilia	Recovered	Cephalea	3250	Daily for 2 days before ALI.	7		
												Metoclopramide	6
												Bisacodyl	6

**Possible (3 to 5)**													

5	M/18	13	5.27/1.24	5.88	M	Fever	Recovered	Cephalea	650	1 day, 11 days before ALI.	5		
												Budesonide	3

6	F/23	13	50.23/2.16	9.23	HC	Fever, rash	Recovered	Cephalea	500	1 day, 2 months before ALI.	4		
								Fever	1950	1 day, 3 months before ALI.			
								Fever	1950	3 days, between day 21 and day 13 before ALI.			
												Carbocysteine	3
												Ambroxol	3
												Benzocaine	3
												Chlorhexidine	3

7	F/35	20	61.10/0.64	22.43	HC	No	Recovered	Dysmenorrhea	650	Once a month for last year till 11 days before ALI.	3	No	-

Alcohol use is expressed as a mean in grams per day in the last year; ALT, alanine aminotransferase and AP, alkaline phosphastase and Bili, bilirubin, expressed in times the upper limit of normal values; outcome at 6 months of follow-up; ALI, acute liver injury; HC, hepatocellular; M, mixed; Tx, liver transplantation.

Six cases were female and the median age was 35 (range 18-85).

The median daily dose of paracetamol was 650 mg (range 650-3,250). The median duration of the exposure period, i.e. the period between the first and the last day of use was 10 days (range 1-77) in five patients. The other two patients had taken the drug for at least one year. The median alcohol consumption was 13.43 g per day (range 0-42.67).

In six patients the pattern of liver injury was hepatocellular. Five cases showed symptoms of hypersensitivity (fever, rash, eosinophilia, thrombocytopenia or arthralgia). All cases except one were taking other drugs. Hypersensitivity reactions have been described for metamizol, dextromethorphan, indapamide, indomethacin, corticosteroids, metoclopramide, bisacodyl, budesonide, carbocysteine, ambroxol, benzocaine, chlorhexidine [20].

After 6 months of follow-up, five patients recovered completely (median of 3.7 months; range 1.27-21.8), one had undergone a liver transplant, while another had not fully recovered.

The estimated population incidence of ALI related to the use of paracetamol at therapeutic dosages based on the 7 cases classified as probable or possible was 0.4 per 10^6 ^inhabitants older than 15 years of age and per year (95%CI, 0.2-0.8) and of 10 per 10^6 ^paracetamol users-year (95% CI 4.3-19.4).

## Discussion

To our knowledge, this is the largest prospective series describing cases of severe ALI in patients exposed to therapeutic dosages of paracetamol that have ever been published.

In 7 out of 26 patients with ALI previously exposed to paracetamol, the RUCAM causality algorithm score for the drug was higher than that for other concomitant medications. Out of these seven cases the association with paracetamol was classified as probable in four of these cases. Six of these seven patients had a hepatocellular pattern of liver injury which is typical of paracetamol overdose and six were female [21]. Of note, most of these cases had accompanying hypersensitivity symptoms.

Although paracetamol was classified as a probable or possible cause of ALI in the 19 remaining cases, these patients had also taken other known hepatotoxic drugs which achieved higher scores than that of paracetamol. However, a contributory role of paracetamol in liver injury in these cases cannot be ruled out [22].

Paracetamol in high single doses (typically 15 g or more) causes liver injury through a toxic metabolite, NAPQI (N-acetyl-p-benzoquinone imine). Alcohol consumption and possibly starvation induce cytocrome P-450 and therefore increase NAPQI synthesis. These factors also contribute to glutathione depletion, [23,24] thus enhancing paracetamol hepatotoxicity. Therefore, heavy drinkers would be at high risk of liver toxicity with paracetamol taken in relatively high doses [8,9,21,25-27]. Acute liver failure has also been reported after doses up to 5 g per day, particularly in alcoholic patients, as well as in fasting patients and patients with underlying liver disease [8,28-30]. Those seven cases reported low alcohol consumption, except case number 1. This patient underwent a liver transplant, and we cannot rule out that paracetamol probably enhanced alcohol toxicity. No patient turned up with a wasting disease or with signs of starvation.

A systematic review of the occurrence of acute liver failure associated with paracetamol in therapeutic dosages reported a median duration of exposure of 6 days in the review of RCTs and 10 days in the retrospective studies. These figures compare well with the median duration of the exposure among five cases with short use of paracetamol (10 days). In addition, 26% of the patients in the systematic review had taken another drug with a causality score higher than that of paracetamol, [10] compared to 73% in our series. However, it should be pointed out that our study was specially designed to collect drug exposures including over the counter drugs and herbs in a rigorous way.

Liver injury after therapeutic dosages of paracetamol has been described in several case reports, but the patients had concurrent potentially contributing conditions, such as asymptomatic HIV infection, hepatitis B or hepatitis C virus infection, heavy alcohol consumption (more than 50 g per day), and/or fasting and nutritional impairment [31-36]. In our study patients with these conditions were excluded. In a randomized controlled trial, 31% - 44% of healthy young participants experienced an ALT level greater than 3 times the upper limit of the normal range (ULN) and more than 19% of participants experienced ALT more than five times the ULN during treatment with paracetamol at a dose of 4 g per day for 14 days, although none developed symptoms or laboratory evidence of hepatic failure [11].

Hypersensitivity to paracetamol is rare, although a few cases of paracetamol-induced idiosyncratic hepatic injury [37-39] have been described. One case recurred on drug rechallenge, and showed symptoms of hypersensitivity [31]. Paracetamol seldom produces allergic skin reactions or respiratory symptoms, [40,41] and anaphylactic shock also occurs very rarely [42]. Thrombocytopenia related to paracetamol has also been described even though in one case it can be explained by the use of other drugs (non steroidal anti inflammatory drugs) taken by the patient [43]. In our series, three of the four patients with the highest causality scores for paracetamol had symptoms of hypersensitivity, suggesting that an immunological mechanism could be involved. This mechanism may explain the presence of ALI in cases with a short period of exposure.

The population-based incidence of ALI related to paracetamol at therapeutic dosages is low, less than one case per 10^6 ^inhabitants per year in our study area. Paracetamol use at therapeutic dosages may be considered safe enough regarding its potential liver toxicity. However, considering the high amount of paracetamol consumption [44] in developed countries it is important to take into account this data in terms of its public health impact.

Our study contributes to completing the information about drug-induced liver injury, especially about liver toxicity and paracetamol at therapeutic dosages, a topic that has not been studied from a prospective design up to now as far as we know. In fact its incidence is unknown and we provide an estimation where paracetamol is one of the most widely used analgesics. There has also been some controversy about its importance since the publication of a RCT [11] where the increase in transaminases seems more frequent than had previously been thought.

Our study has several strengths. Stringent clinical inclusion criteria were applied, case ascertainment was prospective, and the case definition depended on strict clinical criteria rather than on exposure to any particular drug. Additionally, intensive systematic ascertainment of all patients fulfilling the study criteria was performed within the study area. The exclusion of patients with alcohol consumption greater than 50 g per day and/or with other possible causes of liver injury (the principal causes were serologically proven viral hepatitis, alcoholic liver disease, processes causing obstruction of bile flow and drug overdoses) allows us to focus on a more selected population where other causes of ALI have been discarded. In order to avoid selection bias, only serious forms of acute liver disease, usually with jaundice, were included. In addition, details on previous use of medicines (including over the counter drugs and herbs) and alcohol consumption were carefully recorded by asking patients using a detailed structured questionnaire about symptoms, specific indications that covered the use of medicines most frequently associated with liver disease, and by showing them pictures of the major medications of interest. Patients with other known causes of liver disease were excluded. A standardized method of causality assessment was used which may overcome some of the disadvantages of expert opinions in causality assessment of drug reactions.

Our study also has several limitations. Causality assessment was based on algorithms which are relatively simple to apply but where the weighting of each criterion can be somewhat arbitrary. Even though the RUCAM scale is considered at present as the best method for assessing causality in drug-induced liver disease, [45,46] the scale has some limitations, such as the poor definition of the items to be answered and the scarce diagnostic capability when two drugs are administered at the same time. This algorithm awards higher scores to the known hepatotoxic medications, and therefore it may tend to underestimate the causative role of any medication previously unknown to be hepatotoxic. However, not only recently marketed but also old drugs still not incriminated in hepatotoxicity could be to blame [45]. On the other hand, the RUCAM scale does not take into account symptoms of hypersensitivity, while nearly one third of acute hepatic drug reactions are mediated by immunoallergic mechanisms. In addition, very late onset of ALI is awarded lower scores, while several drugs are known to cause liver injury after latency periods of more than 90 days [47]. Finally, the algorithm does not take into account the indication of the suspected medication, which in turn may actually have been prescribed to alleviate early symptoms of liver disease.

## Conclusions

The data from the present case series using detailed clinical and laboratory information indicate that paracetamol in therapeutic dosages may be considered a factor in the causality assessment in patients with liver injury in non-alcoholic individuals,(even if the estimated incidence appears to be low). However, the diagnosis of acute liver injury and the causality assessment to a particular drug remains a difficult task. Liver injury shows a hepatocellular pattern and concomitant symptoms of hypersensitivity in a proportion of cases suggest that the mechanism could be immune mediated. The threshold dose for paracetamol toxicity may vary among individuals, and it may depend on genetic as well as environmental factors. Further studies of the intrinsic mechanisms of hepatotoxicity and the research in biomarkers are needed for a better clinical management of those patients. The estimated population incidence and the incidence in paracetamol users were low and paracetamol use at therapeutic dosages may be considered safe enough regarding its potential liver toxicity.

## List of abbreviations

ALI: acute liver injury; ALT: alanine aminotransferase; RR: relative risk; AP: alkaline phosphatase; CIOMS: Concil for International Organizations of Medical Sciences; RUCAM: Roussel Uclaf Causality Assessment Method; NAPQI: N-acetyl-p-benzoquinone imine; ULN: upper limit of normal.

## Competing interests

The authors declare that they have no competing interests.

## Authors' contributions

MS participated in the data analysis, interpretation of data and writing the paper. LI participated in the conception and design, interpretation of data, and writing the paper. EP participated in the conception and design, data collection, data analysis, and revising it critically for important intellectual content. XV participated in the conception and design, interpretation of data, data analysis and drafting the article. JRL participated in the conception and design, interpretation of data, and drafting the article, and revising it critically for important intellectual content. All other authors participated in the case detection, interpretation of data, and revising it critically for important intellectual content. All authors read and approved the final manuscript.

## Pre-publication history

The pre-publication history for this paper can be accessed here:

http://www.biomedcentral.com/1471-230X/11/80/prepub

## References

[B1] Larson AM Polson J Fontana RJ Davern TJ Lalani E Hynan LS Reisch JS Schiodt FV Ostapowicz G Shakil AO Lee WM Acute Liver Failure Study Group Acetaminophen-induced acute liver failure: results of a United States multicenter, prospective study Hepatology 2005 42 1364 72 10.1002/hep.20948 16317692

[B2] Bower WA Johns M Margolis HS Williams IT Bell BP Population-based surveillance for acute liver failure Am J Gastroenterol 2007 102 1 5 10.1111/j.1572-0241.2007.01057.x 17608778

[B3] Myers RP Li B Shaheen AA Emergency department visits for acetaminophen overdose: a Canadian population-based epidemiologic study (1997-2002) CJEM 2007 9 267 74 17626691 10.1017/s1481803500015153

[B4] Lee WM Squires RH Jr Nyberg SL Doo E Hoofnagle JH Acute liver failure: summary of a workshop Hepatology 2008 47 1401 15 18318440 10.1002/hep.22177 PMC3381946

[B5] Moling O Cairon E Rimenti G Rizza F Pristerá R Mian P Severe hepatotoxicity after therapeutic doses of acetaminophen Clin Ther 2006 28 755 60 10.1016/j.clinthera.2006.05.002 16861097

[B6] Ostapowicz G Fontana RJ Schiodt FV Larson A Davern TJ Han SHB Results of a prospective study of acute liver failure at 17 tertiary care centers in the United States Ann Intern Med 2002 137 947 54 12484709 10.7326/0003-4819-137-12-200212170-00007

[B7] Escorsell A Mas A de la Mata M Spanish Group for the Study of Acute Liver Failure Acute liver failure in Spain: analysis of 267 cases Liver Transpl 2007 13 1389 95 10.1002/lt.21119 17370334

[B8] Prescott LF Therapeutic misadventure with paracetamol: fact or fiction Am J Ther 2000 7 99 114 10.1097/00045391-200007020-00007 11319578

[B9] Graham GG Scott KF Day RO Tolerability of paracetamol Drug Safety 2005 28 227 40 10.2165/00002018-200528030-00004 15733027

[B10] Dart RC Bailey E Does therapeutic use of acetaminophen cause acute liver failure? Pharmacotherapy 2007 27 1219 30 10.1592/phco.27.9.1219 17723075

[B11] Watkins PB Kaplowitz N Slattery JT Colonese CR Colucci SV Stewart PW Harris SC Aminotransferase elevations in healthy adults receiving 4 grams of acetaminophen daily JAMA 2006 296 87 93 10.1001/jama.296.1.87 16820551

[B12] Sabaté M Ibáñez L Pérez E Vidal X Buti M Xiol X Mas A Guarner C Forne M Sola R Castellote J Rigau J Laporte JR Risk of acute liver injury associated with the use of drugs: a multicentre population survey Aliment Pharmacol Ther 2007 25 1401 09 10.1111/j.1365-2036.2007.03338.x 17539979

[B13] Benichou C Criteria of drug-induced liver disease. Report of International Consensus Meeting J Hepatol 1990 11 272 76 2254635 10.1016/0168-8278(90)90124-a

[B14] Ibáñez L Pérez E Vidal X Laporte JR Grup d'Estudi Multicènteric d'Hepatotoxicitat Aguda de Barcelona (GEMHAB) Prospective surveillance of acute serious liver disease unrelated to infectious, obstructive, or metabolic diseases: epidemiological and clinical features, and exposure to drugs J Hepatol 2002 37 592 600 10.1016/S0168-8278(02)00231-3 12399224

[B15] Danan G Benichou C Causality assessment of adverse reactions to drugs - I. A novel method based on the conclusions of internal consensus meetings: application to drug-induced liver injuries J Clin Epidemiol 1993 46 1323 30 10.1016/0895-4356(93)90101-6 8229110

[B16] Lucena MI Camargo R Andrade RJ Perez-Sanchez CJ Sanchez de la Cuesta Comparison of two clinical scales for causality assessment in hepatotoxicity Hepatology 2001 33 123 30 10.1053/jhep.2001.20645 11124828

[B17] Lee WM Assessing causality in drug-induced liver injury J Hepatol 2000 33 1003 05 10.1016/S0168-8278(00)80136-1 11131436

[B18] García-Cortés M Lucena MI Pachkoria K Borraz Y Hidalgo R Andrade RJ Spanish Group for the Study of Drug-induced Liver Disease (Grupo de Estudio para las Hepatopatías Asociadas a Medicamentos) Evaluation of Naranjo adverse drug reactions probability scale in causality assessment of drug-induced liver injury Aliment Pharmacol Ther 2008 27 780 89 10.1111/j.1365-2036.2008.03655.x 18284654

[B19] Benichou C Danan G Flahault A Causality assessment of adverse reactions to drugs - II. An original model for validation of drug causality assessment methods: case reports with positive rechallenge J Clin Epidemiol 1993 46 1331 36 10.1016/0895-4356(93)90102-7 8229111

[B20] BOT y Atención Farmacéutica (CD-ROM) Consejo General de Colegios Oficiales de Farmacéuticos Versión 4. Madrid 200921197180

[B21] Bromer MQ Black M Acetaminophen hepatotoxicity Clin Liver Dis 2003 7 351 67 10.1016/S1089-3261(03)00025-4 12879988

[B22] Lewis JH Ahmed M Shobassy A Palese C Drug-induced liver disease Curr Opin Gastroenterol 2006 22 223 33 10.1097/01.mog.0000218958.40441.fd 16550036

[B23] McClain CJ Price S Barve S Devalarja R Shedlofsky S Acetaminophen hepatotoxicity: an update Curr Gastroenterol Rep 1999 1 42 49 10.1007/s11894-999-0086-3 10980926

[B24] Leeder JS Pirmohamed M Kaplowitz N, DeLeve LD Anticonvulsant agents Drug-induced liver disease 2003 New York: Marcel Dekker 425 46

[B25] Rumack BH Acetaminophen misconceptions Hepatology 2004 40 10 15 15239079 10.1002/hep.20300

[B26] Kaplowitz N Acetaminophen hepatotoxicity: what do we know, what don't we know, and what do we do next? Hepatology 2004 40 23 26 15239082 10.1002/hep.20312

[B27] Rumack BH Acetaminophen hepatotoxicity: the first 35 years J Toxicol Clin Toxicol 2002 40 3 20 10.1081/CLT-120002882 11990202

[B28] Holtzman JL The effect of alcohol on acetaminophen hepatotoxicity Arch Intern Med 2002 162 1193 10.1001/archinte.162.10.1193 12020193

[B29] Kuffner EK Dart RC Bogdan GM Hill RE Casper E Darton L Effect of maximal daily doses of acetaminophen on the liver of alcoholic patients Arch Intern Med 2001 161 2247 52 10.1001/archinte.161.18.2247 11575982

[B30] Heard K Green JL Bailey JE Bogdan GM Dart RC A randomized trial to determine the change in alanine aminotransferase during 10 days of acetaminophen (acetaminophen) administration in subjects who consume moderate amounts of alcohol Aliment Pharmacol Ther 2007 26 283 90 10.1111/j.1365-2036.2007.03368.x 17593074

[B31] Andrade RJ Lucena MI García-Escaño MD Camargo R Severe idiosyncratic acute hepatic injury caused by paracetamol J Hepatol 1998 28 1078 80 10.1016/S0168-8278(98)80361-9 9672188

[B32] Andrade RJ Lucena MI Fernández-Sánchez MC Peláez G Hepatotoxicidad por paracetamol a dosis terapéuticas Med Clin 1997 109 317 18 9379757

[B33] Andrade RJ Lucena MI Melgarejo F García-Escaño MD Hepatotoxicity caused by isoniazid or paracetamol Gastroenterol Hepatol 1998 21 314 15 9711017

[B34] Bolesta S Haber SL Hepatotoxicity associated with chronic acetaminophen administration patients without risk factors Ann Pharmacother 2002 36 1482 83 10.1345/aph.1A035 11847957

[B35] Moling O Cairon E Rimenti G Rizza F Pristerá R Mian P Severe hepatotoxicity after therapeutic doses of acetaminophen Clin Ther 2006 28 755 60 10.1016/j.clinthera.2006.05.002 16861097

[B36] Kurtovic J Riordan SM Acetaminophen-induced hepatotoxicity at recommended dosage J Intern Med 2003 253 240 43 10.1046/j.1365-2796.2003.01097.x 12542566

[B37] Guérin C Casez JP Vital-Durand D Levrat R Allergy to paracetamol. A case of hepatic and cutaneous involvement Therapie 1984 39 47 49 6235621

[B38] Bacon TH Hole JG North M Burnett I Analgesic efficacy of sustained release paracetamol in patients with osteoarthritis of the knee Br J Clin Pharmacol 2002 53 629 36 10.1046/j.1365-2125.2002.01603.x 12047487 PMC1874331

[B39] Kaplowitz N Drug-induced liver injury Clin Infect Dis 2004 38 S44 S48 10.1086/381446 14986274

[B40] Boussetta K Ponvert C Karila C Bourgeois ML Blic J Scheinmann P Hypersensitivity reactions to paracetamol in children: a study of 25 cases Allergy 2005 60 1174 77 10.1111/j.1398-9995.2005.00855.x 16076304

[B41] de Paramo BJ Gancedo SQ Cuevas M Camo IP Martin JA Cosmes EL Paracetamol (acetaminophen) hypersensitivity Ann Allergy Asthma Immunol 2000 85 508 11 10.1016/S1081-1206(10)62580-X 11152174

[B42] Kvedariene V Bencherioua AM Messaad D Godard P Bousquet J Demoly P The accuracy of the diagnosis of suspected paracetamol (acetaminophen) hypersensitivity: results of a single-blinded trial Clin Exp Allergy 2002 32 1366 69 10.1046/j.1365-2222.2002.01476.x 12220477

[B43] Kenney B Stack G Drug-induced Thrombocytopenia Arch Pathol Lab Med 2009 133 309 314 19195976 10.5858/133.2.309

[B44] Abecia LC García del Pozo J de Abajo FJ Utilización de analgésicos no opioides en España 1992-2006 AGEMED 2009 http://www.aemps.gob.es/medicamentosUsoHumano/observatorio/docs/analgesicos_no_opio.pdf

[B45] Lucena MI García-Cortés M Cueto R Lopez-Duran J Andrade RJ Assessment of drug-induced liver injury in clinical practice Fundam Clin Pharmacol 2008 22 141 58 10.1111/j.1472-8206.2008.00566.x 18353109

[B46] Chalasani N Fontana RJ Bonkovsky HL Watkins PB Davern T Serrano J Yang H Rochon J Drug Induced Liver Injury Network (DILIN) Causes, clinical features, and outcomes from a prospective study of drug-induced liver injury in the United States Gastroenterology 2008 135 1924 34 10.1053/j.gastro.2008.09.011 18955056 PMC3654244

[B47] Shapiro MA Lewis JH Causality assessment of drug-induced hepatotoxicity: promises and pitfalls Clin Liver Dis 2007 11 477 505 10.1016/j.cld.2007.06.003 17723916

